# Efficacy and safety comparison of infrared laser moxibustion and traditional moxibustion in knee osteoarthritis: study protocol for a Zelen-design randomized controlled non-inferiority clinical trial

**DOI:** 10.1186/s13018-023-04408-x

**Published:** 2023-12-02

**Authors:** Zhong-yu Wang, Fang-fang Chen, Jiang-Tao Li, Bai-xiao Zhao, Li Han

**Affiliations:** 1 School of Traditional Chinese Medicine, Beijing University of Chinese Medicine, Beijing, 100010 China; 2 School of Acupuncture-Moxibustion and Tuina, Beijing University of Chinese Medicine, Beijing, 100029 China; 3 Technical Institute of Physics and Chemistry, Chinese Academy of Sciences, Beijing, 100190 China

**Keywords:** Moxibustion, Infrared laser moxibustion, KOA, Non-inferiority, Randomized controlled trial

## Abstract

**Background:**

Knee osteoarthritis (KOA) is the most common chronic degenerative joint disease and places a substantial burden on the public health resources in China. The purpose of this study is to preliminarily evaluate whether infrared laser moxibustion (ILM) is non-inferior to traditional moxibustion (TM) in the treatment of KOA.

**Materials and methods:**

In the designed Zelen-design randomized controlled non-inferiority clinical trial, a total of 74 patients with KOA will be randomly allocated to one of two interventions: ILM treatment or TM treatment. All participants will receive a 6-week treatment and a follow-up 4 weeks after treatment. The primary outcomes will be the mean change in pain scores on the numeric rating scale (NRS) measured at baseline and the end of last treatment at week 6. The secondary outcomes will be the pain scores on the NRS from weeks 1 to 5 after the start of treatment and the changes from baseline to endpoints (weeks 6 and 10) in the Western Ontario and McMaster Universities Osteoarthritis Index (WOMAC), SF-36, knee circumference, and 6-min walking test. In addition, safety assessment will be performed throughout the trial.

**Conclusion:**

The results of our study will help determine whether a 6-week treatment with ILM is non-inferior to TM in patients with KOA, therefore providing evidence to verify if ILM can become a safer alternative for TM in clinical applications in the future.

*Trial registration*: Clinical Trial Registration Platform (ChiCTR2200065264); Pre-results. Registered on 1 November 2022.

**Supplementary Information:**

The online version contains supplementary material available at 10.1186/s13018-023-04408-x.

## Introduction

Osteoarthritis (OA) is one of the most common chronic degenerative joint diseases caused by abnormal metabolism of joint tissues. It usually manifests as early onset pain, followed by stiffness, swelling, injury of periarticular tissue, limited activity and deformity [1, 2], all of which can affect the patient’s quality of life [3, 4]. As one of the primary load-bearing joints, the knee is frequently affected by OA. In China, due to the aging population and economic development that has led more people to seek medical treatment, and the occurrence rate of knee Osteoarthritis (KOA) has been increasing annually from 2008 to 2017. Moreover, KOA is no longer exclusive to the elderlies, an increasing number in the younger population are also suffering from this disease, and all of which yield to a substantial burden on the public health resources in China [5].

In the early stages of KOA, it should be treated conservatively with the primary goal of alleviating joint pain in mild to moderate KOA [6]. Pharmacological therapies including non-steroidal anti-inflammatory drugs, acetaminophen, glucosamine, and chondroitin sulfate only can provide temporary pain relief in addition to having toxic side effects [7, 8]. The recommended safe and long-term measures, which include patient education, physical exercise, and weight management, are mostly preventive and relatively difficult to implement [9, 10].

Traditional moxibustion (TM) is a commonly used alternative therapy for KOA in Asia [11, 12]. The efficacy of TM is considered to result from a combination of factors [13–15], among them, near-infrared (NIR) light is generally thought to play a major role in the biological radiation effect of moxibustion. The NIR radiation can induce active substances to promote the metabolism and thermogenesis of the organs reached by the NIR rays, thereby adjusting the body’s immune and neurological functions by energizing cell metabolism [16–18]. Other studies indicated that photobiomodulation plays an active role in anti-inflammatory and analgesia [19, 20]. In recent years, the Chinese regime has been greatly emphasizing on the integration of traditional Chinese medicine (TCM) instruments with modern science and technology, and some novel moxibustion instruments have been developed to minimize the adverse effects of TM while maintaining its effectiveness [21–24]. However, the currently available instruments have two major problems: (1) the working temperature is much lower than the burning temperature of natural moxa pillars; and (2) the characteristics of moxibustion burning infrared spectrum have not yet compiled a unified standard, so none of these instruments can actualize a high simulation of the natural moxibustion emission spectrum and hindering the therapeutic effects.

For the above reasons, we will set up an infrared emissivity test system to accurately test for a reliable basic data of moxibustion emission spectrum, and then regulate the emission spectrum of the material by regulating the composition structure of infrared ceramics/graphene to realize the coupling between it and the absorption spectrum of human acupoints [25]. On this basis, we use a Mg–ZrO2/ graphene specified composite ceramic block as the key material of emission light source to develop infrared laser moxibustion (ILM). Therefore, we designed a Zelen-design randomized controlled non-inferiority clinical trial to (1) preliminarily investigate whether ILM is non-inferior to TM in reducing pain and improving function in patients with KOA; and (2) provide clinical evidence to verify whether ILM can become a safe and reliable clinical alternative to TM in the future.

## Methods

### Study design

**Fig. 1:**
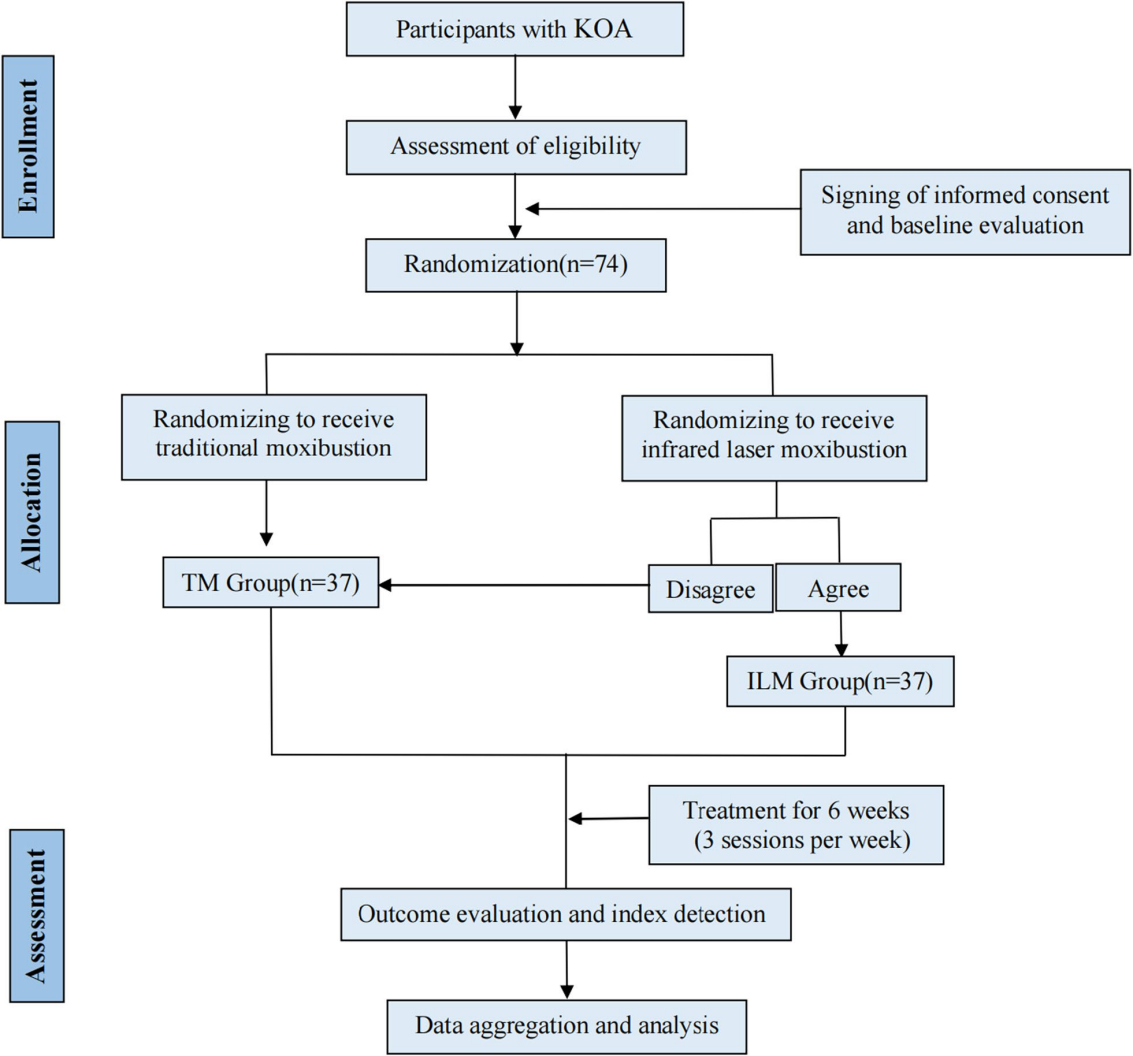
Flow chart *KOA*, knee osteoarthritis; *TM*, traditional moxibustion; *ILM*, Infrared laser moxibustion

The Zelen-design randomized controlled non-inferiority clinical trial will be conducted at Beijing Electric Power Hospital of the State Grid Corporation of China and AMHT Group Aerospace 731 Hospital. The trial protocol is in accordance with the SPIRIT guidelines and the CONSORT guidelines, [26, 27] and the above two checklists are provided as Additional file 1. This trial was approved by the Beijing University of Traditional Chinese Medicine on 17 October 2022 (Ethics Reference No: 2022BZYLL1017). In view of the ethical requirements of current clinical trials, the schedule of recruitment, randomization, informed consent, intervention, and assessment are shown in Fig. 1.

### Trial status

This clinical trial is currently recruiting patients. Enrollment started in November 2022 and is expected to be completed by November 2023.

### Participants

Recruitment will be facilitated by the outpatient departments of the two hospitals and advertising on the We Chat platform. All treatments, assessments, blood tests, and knee X-rays will be provided free of charge. And at the end of the study, we will provide participants with a subsidy based on each patient’s degree of cooperation.

### Inclusion criteria

The inclusion criteria were as follows:

1. Diagnosed with KOA according to the American College of Rheumatology criteria [28].
2. Aged 40–70 years old.
3. Average pain score of 4 or more out of 10 on the numeric rating scale (NRS) [29].
4. Average severity score of 48 or less on the Western Ontario and McMaster Universities Osteoarthritis Index (WOMAC) [30].
5. Standard radiographs with Kellgren–Lawrence (KL) grade II or III [31].
6. Written informed consent.
7. With basic reading and comprehension skills.

Participants who fulfill the above seven criteria can be included in the trial.

### Exclusion criteria

The exclusion criteria were as follows:

1. Received knee surgery in the past six months or waiting for surgery.
2. Received acupuncture treatment for KOA in the past three months.
3. Received an intra-articular injection for KOA in the past six months.
4. Received arthroscopy for KOA in the past year.
5. Had other diseases, infections, or traumatic injuries that could cause knee pain.
6. Had serious cardiovascular and cerebrovascular, respiratory, liver, kidney, metabolic system and other diseases, malignant tumors, mental disorders.
7. Allergic to moxibustion smoke or infrared light.
8. Pregnant or lactating.
9. Received other similar physiotherapy methods in the past month.

Participants who met at least one of the exclusion criteria above will be excluded from the trial.

### Randomization and informed consent

A single-consent Zelen design will be used for allocation and randomization. An independent statistician will generate a random number table using the statistical software SPSS20.0, and the random seed will be set in advance. Each subject’s random number and treatment will be hidden in an opaque envelope. After enrollment assessment, all eligible participants will obtain an envelope in the order of entry.

All participants who meet the inclusion criteria will be randomly assigned to the TM group (the proposed control group) or the ILM group (the proposed treatment group) in a 1:1 ratio. Participants in the ILM group will be asked if they are willing to comply with the current randomized treatment; if they agree, they will be placed in the ILM group; if not, they will be automatically placed in the TM group. Participants in the TM group were directly enrolled without the above questioning process [32, 33], all participants will sign informed consent forms before enrollment [34].

### Blinding

Due to the particularity of ILM treatment, it is difficult to deceive patients and clinicians during the study. However, the outcome evaluators, data collectors, and analysts will know nothing about the grouping and treatment of patients.

### Interventions

Moxibustion treatment in the two groups (approximately 30 min in duration) will be administered three times per week over six weeks, and a follow-up evaluation will be performed at week 10. During the trial period, the participants will be prohibited from receiving relevant treatments, such as knee surgery, diagnostic arthroscopy, intra-articular injection, physiotherapy, acupuncture, massage therapy, and acupoint application.

Both knees of subjects with bilateral osteoarthritis will be treated, but only the most painful knee will be selected for evaluation in each subject. If both knees are equally painful, the knee with the higher KL grade will be chosen [35], if the knee pain and KL ratings are the same on both sides, the knee joints for evaluation will be randomly selected using a random number table.

### Selection of acupoints

**Fig. 2:**
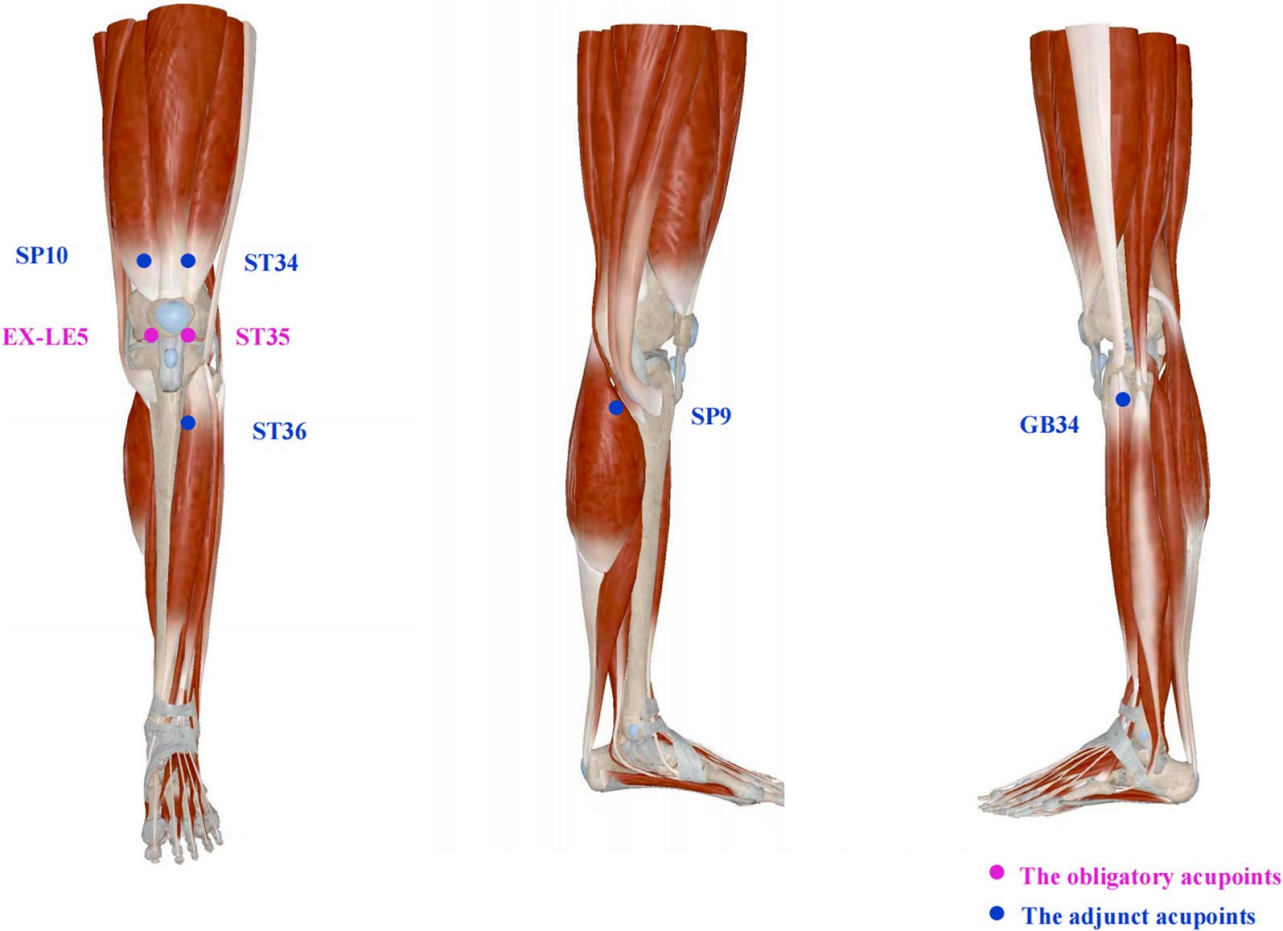
^Locations of acupoints. The round pink marks represent two obligatory acupoints, and the round blue marks represent five adjunet acupoints^

Four acupoints, two obligatory acupoints and two adjunct acupoints will be selected for treatment (Fig. 2). The obligatory acupoints are *dubi* (ST35) and *neixiyan* (EX-LE5), and the adjunct acupoints include *liangqiu* (ST34), *xuehai* (SP10), *yinlingquan* (SP9), *yanglingquan* (GB34), and *zusanli* (ST36). Moxibustion will be applied at two of the five adjunct acupoints according to the location of pain and traditional Chinese medicine (TCM) syndrome differentiation for each person. All acupoints will be located according to the “*WHO Standard Acupuncture Point Locations in the Western Pacific Region*” [36].

### Combined medication

The use of hormones and opioids will be prohibited during the trial. Participants who have taken medication for hypertension, diabetes, or knee pain in the month prior to the start of the trial will continue to take medication during the study but will be required to specify the medication in the case report form (reasons, drug name, type, dose, the time of the intake, and whether the symptoms are relieved after taking the medicine). If the participants are not taking any medications before the trial, they will not take any medications during the study. If the patient develops new symptoms or diseases during the trial, any new drug should be approved by the investigator and should only be used if it does not affect the results of trial.

### ILM group

**Fig. 3:**
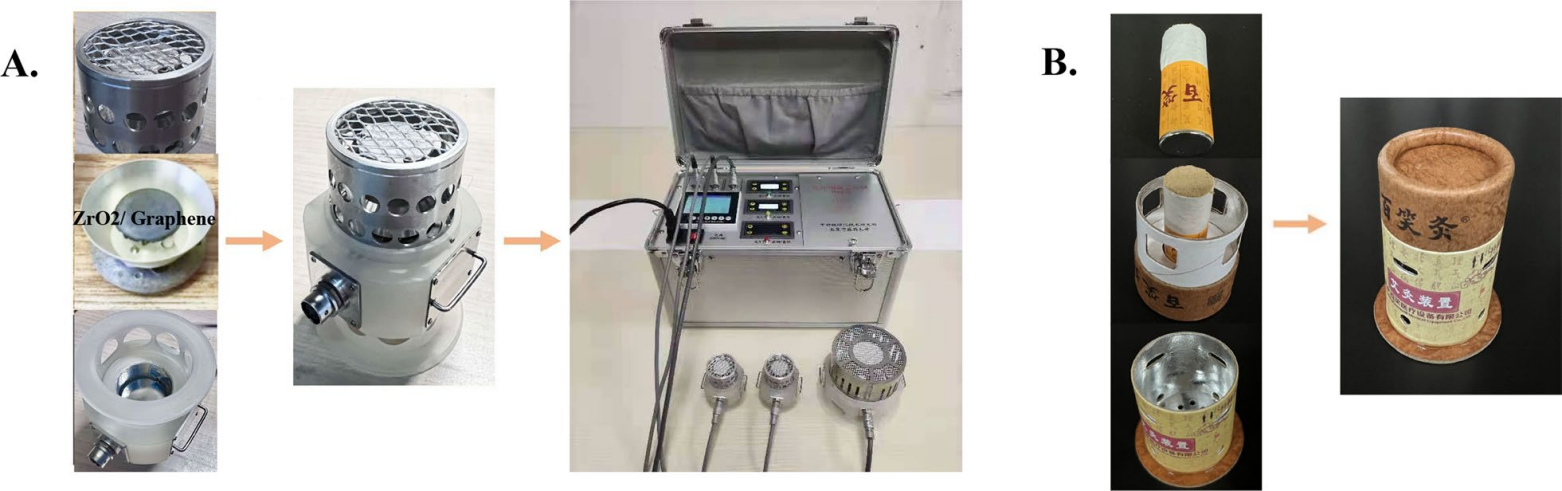
Moxibustion devices. **A** Infrared ceramic graphene moxibustion device. **B** traditional moxibustion device

The device used for treatment in the ILM group will be the ILM shown in Fig. 3 (Chongqing Baixiao Medical Equipment Co. Ltd., model type BX-AJY-001).And the parameters of ILM are shown in Table 1.

**Table 1:** Infrared laser moxibustion parameter

	Parameter
Device	Infrared ceramic graphene moxibustion instrument (Model: BX-AJY-001)
Rated power	600VA
Power condition	a.c.220 V, 50 Hz
Peak wavelength	3.98 µm
Mode	Continuous

Before treatment, the participant will be in the sitting or supine position, and the acupoints will be cleaned and disinfected with cotton balls soaked with 75% alcohol. The researchers will then fix the moxibustion head on the corresponding part of the skin with Velcro and connect the wires. During treatment, the researchers will first turn on the power, start the corresponding moxibustion head on the intelligent terminal. The infrared radiation source of each infrared moxibustion head used in the test will be 5 cm away from the skin. The diameter of the ILM chamber in contact with the skin is 4 cm. Each treatment will last for 30 min.

The design of ILM is aimed at imitating the actual situation and characteristics of TM burning. When the temperature of heat source is 470 °C, the corresponding temperature of human skin surface can reach about 45 °C, which is consistent with TM [25, 37]. If the participant has no obvious thermal sensation or temperature intolerance, the temperature of the infrared radiation source moxibustion can be gradually increased or decreased to a certain extent on the basis of 470 °C. The moxibustion temperature should be tolerable and comfortable for the participants. During treatment, the patient’s general condition will be observed and recorded.

### TM group

The control participants group will receive TM using the Baixiao moxibustion device shown in Fig. 3 (Dimension: 6.4 cm × 3.8 cm, Chongqing Baixiao Medical Equipment Co. Ltd., model type BX-A002).

The researchers involved in the treatment will paste the moxibustion tube onto the acupoints with positioning paper, remove the moxibustion cover, install the moxibustion column, and buckle the column on the moxibustion tube after lighting. The size of the inlet hole of the moxibustion tube can be adjusted by rotating the tube body left and right or pull and push the tube body up and down to moderate the moxibustion temperature. The diameter of the TM chamber in contact with the skin is 3.8 cm. The distance between the heat source and the skin is adjustable, ranging from 1.5 to 4 cm. Each moxibustion column can burn for approximately 30 min. The burning of the moxibustion column is indicated by the disappearance of the thermal sensation and the cooling of the wall of the moxibustion device.

### Outcomes

The assessment schedule is presented in Table 1.

#### Primary outcome

The primary outcome, to be assessed at the end of the last treatment at week 6, is the change in pain from baseline using the NRS index [38]. The NRS index typically uses a 0–10 range instead of words to represent the degree of pain experienced by the subject: 0 indicates no pain; 1–3 indicates mild pain (occasional pain that generally does not interfere with walking); 4–6 indicates moderate pain (frequent pain that interferes with walking); 7–9 indicates severe pain (sustained pain that seriously interferes with or prevents walking); and 10 indicates the worst/overwhelming pain. At the time of the evaluation, participants will be asked to mark the number on the scale that best represents their pain level in the last 24 h.

#### Secondary outcomes

The NRS pain score will also be measured at the end of the last treatment each week (weeks 1, 2, 3, 4, and 5). Other secondary outcomes will include the changes in WOMAC, SF-36, knee circumference, and 6-min walking test (6MWT) from baseline to endpoints (weeks 6 and 10):

1. WOMAC: This scale evaluates the comprehensive condition of the knee joint from three aspects: knee pain, stiffness, and daily functional activities. The WOMAC scale contains 24 items, with four points for each item. A higher score signifies worse knee function. The Chinese version of this scale has good reliability and validity [39].
2. SF-36: This scale consists of 36 items, including eight fields of physical function, arole physical, physical pain, general health status, vitality, social function, arole emotional, and mental health, and is used to assess the health-related quality of life of the subject. A higher score indicates better health-related quality of life [40, 41].
3. Knee circumference: The degree of swelling in the subject’s knee was assessed by measuring the knee circumference (i.e., the leg circumference at the center of the upper and lower edges of the patella) in millimeters. A uniform tape measure will be used to measure different patients [42].
4. 6MWT: Before the test, researchers will draw a 30-m straight line on flat ground and mark both ends of the line. During the test, the participants will walk back and forth in a straight line at their own speed. The researchers will give the correct time every two minutes and calculate the distance walked by the participant at the end of six minutes. Potential discomfort such as fatigue, dizziness, angina pectoris, dyspnea and cold sweat will be recorded during the trial. The trial will be suspended or discontinued if the patient cannot complete it. During the test, participants will be monitored with a unified wristwatch for basic indicators such as heart rate and blood oxygen. The Borg score of dyspnea and fatigue and other data will be recorded before and after the test (Table 2) [43].

**Table 2:**
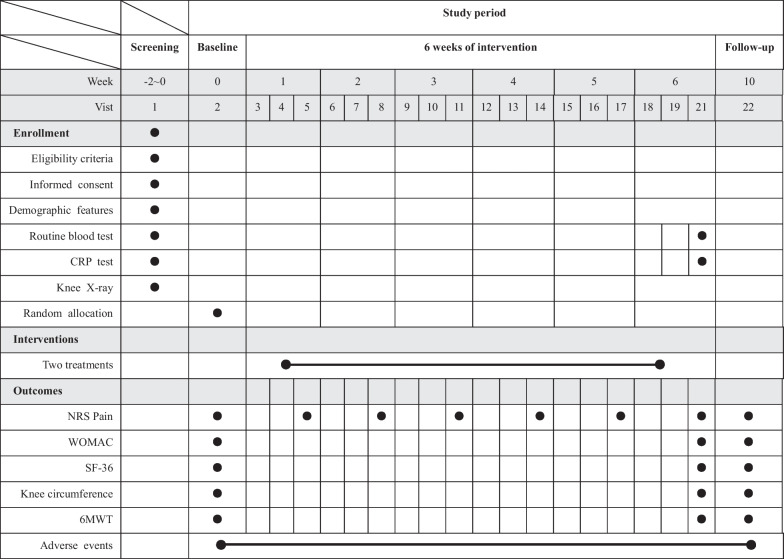
Schedule of enrolment, interventions and assessments

*CRP test,* C-reactive protein test; *WOMAC*, Western Ontario and McMaster Universities Osteoarthritis Index; *SF-36*, 36-item Short-Form; *6MWT*, 6-min walking test

### Safety assessment

The safety assessment will primarily include the recording of adverse events (AEs) and serious AEs, laboratory analyses, and monitoring throughout the exercise test. AEs occurring during treatment and follow-up will be recorded by the investigator on AE questionnaires within 24 h of their occurrence. In addition to recording the time, severity, reaction, treatment, and prognosis of the AEs, the questionnaire will also focus on recording the potential relationship between AEs and TM or ILM treatment. The most common treatment-related AEs include skin redness, pruritus, scalding, blisters, and dizziness. If the AE is severe, the patient will be suspended or withdrawn from the study and receive corresponding medical care or compensation. In addition, blood routine and C-reactive protein (CRP) measurement will be conducted before and after treatment. Lastly, a uniform sports wristwatch will be used to monitor the participants’ heart rates during the 6-min walk test.

### Study quality control and data management

The trial protocol for this clinical study has been reviewed and confirmed by methodological and statistical experts in relevant fields, and the trial will be carried out in strict accordance with this protocol. Any changes to the protocol will need to be approved by the Ethics Committee.

Before the study begins, all researchers involved in the treatment will be instructed and trained in the standardized operating procedures. The training content will include the implementation steps and methods of the project, the operation methods of traditional moxibustion and the infrared ceramic graphene moxibustion device, and the management of adverse events. To ensure the quality and integrity of the trial, each clinical trial site will have a clinical supervisor specializing in acupuncture and moxibustion who will supervise the entire process.

The original data from this project will be recorded in detail in the individual case report forms (CRFs), informed consent form, clinical medical records, laboratory test sheets, and imaging data, all of which are traceable. The investigator will manually fill out the CRFs for all patients. The investigator will indicate their name and the date, periodically collect the data. Any changes made to the data and reasons for any missing data will also be indicated in the notes.

Three months after the publication of the clinical study, the original data will be shared through the ResMan platform of the Chinese Clinical Trial Registry (http://www.medresman.org.cn).

### Sample size calculation

This study is a randomized controlled non-inferior trial. Based on the literature, the smallest clinically significant difference in the pain NRS is 2 points [44–46]. Thus, we set the non-inferiority margin to − 2. The NRS score of the subjects after treatment was taken as the main outcome index. According to the results of similar studies, [47] the average NRS score of the experimental group was 40.53 ± 26.63, and that of the control group was 41.58 ± 15.07. So we used PASS 15 to calculate the sample size (29 participants in each group), *α* = 0.025, 1 − *β* = 80%. Considering that a maximum of 25% dropped out during the trial, we ultimately plan to recruit 74 participants (37 in each group) to ensure the target sample size is obtained. The calculation results of sample size are provided as Additional file 2.

### Statistical analysis

Statistical analysis will be undertaken using both intention-to-treat (ITT) and per-protocol (PP) analyses. For the ITT analysis, all patients who undergo the Zelen-type randomization and received at least one treatment will be analyzed. Primary PP data analysis will also be undertaken for all patients who have high compliance (patient compliance rates equal to or greater than 80%) and do not use drugs or treatments prohibited by the protocol during treatment.

The statistical analysis will take the form of a non-inferiority test. The margin of non-inferiority is -2. For the main outcome, we will calculate the 95% confidence interval for the difference in NRS pain score between the treatment group and the control group. If the lower limit is greater than -2, non-inferiority will be considered valid.

For continuous data, mean ± standard deviation will be used to describe data that conforms to a normal distribution, whereas non-normally distributed continuous data will be described as the median (interquartile spacing). Where the data conforms to a normal distribution, independent sample t-test will be used for comparison between the two groups with homogeneity of variance; otherwise, a t-test will be used with heterogeneity of variance. Wilcoxon rank-sum test will be used if data does not conform to a normal distribution. Categorical data will be represented by frequency or percentage and compared using chi-square test or Fisher exact test. Missing data will be replaced according to the last observation carried forward method. The 95% confidence interval will be calculated, and *P* < 0.05 will be considered to indicate a significant difference. All statistical analysis will be performed using SPSS software (version 20.0).

## Discussion

In recent years, as the state attaches great importance to the scientific research and innovation of the TCM industry, the combination of TCM instruments and modern science and technology has become increasingly close, which has become a new direction for the development of TCM instruments. However, the transformation of TCM needles and instruments mostly stays in the original appearance, rarely using new technology, new materials and upgrading its core technology. Modern technologies such as infrared, laser and acupoint stimulation have been widely used in the research and development of TCM instruments, but many of them have a single stimulation mode, which is easy to cause body tolerance and cannot achieve the effect of TM therapy. For the imitation moxibustion instruments on the market, the accurate simulation of TM cannot be achieved [48]. Some of them are different from traditional moxibustion in terms of shape, size and heating area [49], some simply simulate the thermal effect of moxibustion sticks, lack of discussion and application of other functional factors [21–23]. At the same time, due to the differences in moxibustion materials, amount, application methods, burning conditions and the performance of infrared spectroscopy instruments for measurement over the past years, it is difficult to form a unified standard for the characteristics of moxibustion infrared spectrum, let alone realize accurate simulation of it [24]. Therefore, making full use of modern sensing technology, artificial intelligence, biophysics and other new technologies, infrared ceramics/graphene and other new materials, breaking through technical bottlenecks, and establishing a common key technology platform based on the principles of TCM diagnosis and treatment are the key breakthroughs in the current research and development of TCM medical devices.

Based on the above reasons, we have realized the full band infrared emissivity test of moxibustion in the early stage and developed ILM which can realize accurate simulation of the temperature and emission spectrum of moxibustion combustion (the internal temperature is 736 °C; the external working temperature is about 470 °C; the wavelength corresponding to the peak of the emission spectrum is 3.98 μm) and effectively makes up for the defects of TM and existing moxibustion instruments [25, 37].

This study will be the first clinical trial to evaluate infrared ceramic/graphene laser moxibustion for the treatment of KOA. The study will test the hypothesis that ILM is not inferior to TM for the treatment of KOA. The Zelen-design adopted in this study is consistent with the real diagnosis and treatment environment, thereby minimizing the bias inherent in the traditional design of randomized controlled trials. During the trial, the willingness of patients or their guardians receive different treatments will be considered to help ensure the compliance of patients and the smooth conduct of the trial.

The primary priority in the treatment of KOA is pain relief followed by maintaining normal knee function and improving quality of life; thus, we chose pain on the NRS as the primary outcome and WOMAC, SF-36, knee circumference, and 6MWT as the secondary outcomes. For the safety evaluation, in addition to recording AEs, we will also conduct blood routine and CRP detection before and after the trial. CRP is one of the most commonly used laboratory indicators of systemic inflammatory diseases. The expression of CRP increases in patients with OA and is closely related to the progression and prognosis of the disease [50, 51].

The proposed study has some limitations that should be acknowledged. First, this is a non-inferiority trail; thus, compared with superiority trials, some participants may have greater difficulty adhering to the protocol for their treatment. This may result in incorrectly rejecting the null hypothesis [50]. For this reason, the Zelen-design was used to improve patient compliance. Second, it is difficult to blind the researchers involved in the assignment and treatment due to the differences in the nature of the treatments. Therefore, to reduce the risk of bias, the study evaluation will be conducted by people not involved in assignment or treatment. Third, this will be a small-scale trial with a small sample size. Finally, this study will primarily use subjective scales to evaluate knee joint pain and function; objective physiological indicators are lacking.

The results of our study will help preliminarily determine whether the infrared ceramic graphene moxibustion device is non-inferior to traditional moxibustion in alleviating knee pain and improving related function in KOA patients, therefore providing a suitable alternative for TM in clinical practice. In the future, based on the results of this exploratory study, we hope to design and carry out a multicenter, large-sample, randomized controlled clinical trial with a longer follow-up period to confirm the results and enhance the generalizability of the findings.

### Supplementary Information

**Additional file 1**. CONSORT Checklist and SPIRIT Checklist.

**Additional file 2**. Sample size calculation.

## Data Availability

The original data will be shared through the ResMan platform of the Chinese Clinical Trial Registry, http://www.medresman.org.cn. 3 months after the publication of the clinical study.

## Abbreviations

KOA: Knee osteoarthritis
OA: Osteoarthritis
ILM: Infrared laser moxibustion
TM: Traditional moxibustion
NRS: Numerical rating scale
WOMAC: Western Ontario and McMaster Universities Osteoarthritis index
SF-36: 36-Item short for health survey
6MWT: 6-Min walking test
ITT: Intention-to-treat
TCM: Traditional Chinese medicine
CRF: Case report form
PP: Per-protocol
LOCF: Last observation carried forward
CIs: Confidence intervals
CRP: C-reactive protein
