# Supplementary material for: Efficacy and safety comparison of infrared laser moxibustion and traditional moxibustion in knee osteoarthritis: study protocol for a Zelen-design randomized controlled non-inferiority clinical trial
Source: J Orthop Surg Res. 2023 Dec 2;18:922. doi: 10.1186/s13018-023-04408-x (PMC10693696; doi:10.1186/s13018-023-04408-x)
Supplement: Supplementary file 1 — Additional file 1. CONSORT Checklist and SPIRIT Checklist. [file 13018_2023_4408_MOESM1_ESM.pdf]

STANDARD PROTOCOL ITEMS: RECOMMENDATIONS FOR INTERVENTIONAL TRIALS

SPIRIT 2013 Checklist: Recommended items to address in a clinical trial protocol and related documents*

Section/item	Item No	Description	Reported on Page Number/Line Number	Reported on Section/Paragraph
**Administrative information**				
Title	1	Descriptive title identifying the study design, population, interventions, and, if applicable, trial acronym	Page 1/Line 1-4	Title
Trial registration	2a	Trial identifier and registry name. If not yet registered, name of intended registry	Page 3/Line 3-4	Trial registration Section
	2b	All items from the World Health Organization Trial Registration Data Set	Page 16/Line 5- 7	Study quality control and data management
Protocol version	3	Date and version identifier	In the header of all the pages.	In the header of all the pages.
Funding	4	Sources and types of financial, material, and other support	Page 23/Line 9-11	Funding
Roles and responsibilities	5a	Names, affiliations, and roles of protocol contributors	Page 1/Line 5-19	Authors Section
	5b	Name and contact information for the trial sponsor	Page 23/Line 12-15	Author contributions
	5c	Role of study sponsor and funders, if any, in study design; collection, management, analysis, and interpretation of data; writing of the report; and the decision to submit the report for publication, including whether they will have ultimate authority over any of these activities	Page 23/Line 12-15	Author contributions
	5d	Composition, roles, and responsibilities of the coordinating centre, steering committee, endpoint adjudication committee, data management team, and other individuals or groups overseeing the trial, if applicable (see Item 21a for data monitoring committee)	N/A	N/A
**Introduction**				
Background and rationale	6a	Description of research question and justification for undertaking the trial, including summary of relevant studies (published and unpublished) examining benefits and harms for each intervention	Page 3/Line 8 - Page 5/Line 13	Introduction
	6b	Explanation for choice of comparators	Page 3/Line 8 - Page 5/Line 13	Introduction
Objectives	7	Specific objectives or hypotheses	Page 5/Line 3 - 13	Introduction

Trial design	8	Description of trial design including type of trial (eg, parallel group, crossover, factorial, single group), allocation ratio, and framework (eg, superiority, equivalence, noninferiority, exploratory)	Page5/Line 16 - Page 6/Line 5	Study design
**Methods: Participants, interventions, and outcomes**				
Study setting	9	Description of study settings (eg, community clinic, academic hospital) and list of countries where data will be collected. Reference to where list of study sites can be obtained	Page5/Line 16 - Page 6/Line 5	Study design
Eligibility criteria	10	Inclusion and exclusion criteria for participants. If applicable, eligibility criteria for study centres and individuals who will perform the interventions (eg, surgeons, psychotherapists)	Page 7/Line5 - Page 8/Line 10	Inclusion criteria and Exclusion criteria
Interventions	11a	Interventions for each group with sufficient detail to allow replication, including how and when they will be administered	Page 9/Line 10 - Page 13/Line 3	Interventions
	11b	Criteria for discontinuing or modifying allocated interventions for a given trial participant (eg, drug dose change in response to harms, participant request, or improving/worsening disease)	Page 15/Line 9- Page 16/Line 6	Safety assessment
	11c	Strategies to improve adherence to intervention protocols, and any procedures for monitoring adherence (eg, drug tablet return, laboratory tests)	Page 7/Line 1-4; Page 8/Line 10- Page 9/Line 3	Participants ; Randomization and informed consent
	11d	Relevant concomitant care and interventions that are permitted or prohibited during the trial	Page 9/Line 11- 16	Intervention
Outcomes	12	Primary, secondary, and other outcomes, including the specific measurement variable (eg, systolic blood pressure), analysis metric (eg, change from baseline, final value, time to event), method of aggregation (eg, median, proportion), and time point for each outcome. Explanation of the clinical relevance of chosen efficacy and harm outcomes is strongly recommended	Page 13/Line 4 - Page 15/Line 8	Outcomes
Participant timeline	13	Time schedule of enrolment, interventions (including any run-ins and washouts), assessments, and visits for participants. A schematic diagram is highly recommended (see Figure)	Table 2	Table 2
Sample size	14	Estimated number of participants needed to achieve study objectives and how it was determined, including clinical and statistical assumptions supporting any sample size calculations	Page 17/Line 9 -19	Sample size caculation
Recruitment	15	Strategies for achieving adequate participant enrolment to reach target sample size	Page 8/Line 10- Page 9/Line 3; Page5/Line 16 - Page 6/Line 5	Randomization and informed consent; Study design
**Methods: Assignment of interventions (for controlled trials)**				
Allocation:				
Sequence generation	16a	Method of generating the allocation sequence (eg, computer-generated random numbers), and list of any factors for stratification. To reduce predictability of a random sequence, details of any planned restriction (eg, blocking) should be provided in a separate document that is unavailable to those who enrol participants or assign interventions	Page 8/Line 10- Page 9/Line 3	Randomization and informed consent
Allocation concealment mechanism	16b	Mechanism of implementing the allocation sequence (eg, central telephone; sequentially numbered, opaque, sealed envelopes), describing any steps to conceal the sequence until interventions are assigned	Page 8/Line 10- Page 9/Line 3	Randomization and informed consent

Implementation	16c	Who will generate the allocation sequence, who will enrol participants, and who will assign participants to interventions	Page 8/Line 10- Page 9/Line 3	Randomization and informed consent
Blinding (masking)	17a	Who will be blinded after assignment to interventions (eg, trial participants, care providers, outcome assessors, data analysts), and how	Page 9/Line 4-7	Blinding
	17b	If blinded, circumstances under which unblinding is permissible, and procedure for revealing a participant's allocated intervention during the trial	N/ A	N/ A
**Methods: Data collection, management, and analysis**				
Data collection methods	18a	Plans for assessment and collection of outcome, baseline, and other trial data, including any related processes to promote data quality (eg, duplicate measurements, training of assessors) and a description of study instruments (eg, questionnaires, laboratory tests) along with their reliability and validity, if known. Reference to where data collection forms can be found, if not in the protocol	Page 16/Line 8- Page 17/Line 7	Study quality control and data management
	18b	Plans to promote participant retention and complete follow-up, including list of any outcome data to be collected for participants who discontinue or deviate from intervention protocols	Page 7/Line 1-4; Page 8/Line 10- Page 9/Line 3	Participants ; Randomization and informed consent
Data management	19	Plans for data entry, coding, security, and storage, including any related processes to promote data quality (eg, double data entry; range checks for data values). Reference to where details of data management procedures can be found, if not in the protocol	Page 16/Line 8- Page 17/Line 7	Study quality control and data management
Statistical methods	20a	Statistical methods for analysing primary and secondary outcomes. Reference to where other details of the statistical analysis plan can be found, if not in the protocol	Page 17/ Line 1 - Page 18/ Line 2	Statistical analysis
	20b	Methods for any additional analyses (eg, subgroup and adjusted analyses)	N/ A	N/ A
	20c	Definition of analysis population relating to protocol non-adherence (eg, as randomised analysis), and any statistical methods to handle missing data (eg, multiple imputation)	Page 18/Line 19-20	Statistical analysis
**Methods: Monitoring**				
Data monitoring	21a	Composition of data monitoring committee (DMC); summary of its role and reporting structure; statement of whether it is independent from the sponsor and competing interests; and reference to where further details about its charter can be found, if not in the protocol. Alternatively, an explanation of why a DMC is not needed	N/ A	N/ A
	21b	Description of any interim analyses and stopping guidelines, including who will have access to these interim results and make the final decision to terminate the trial	N/ A	N/ A
Harms	22	Plans for collecting, assessing, reporting, and managing solicited and spontaneously reported adverse events and other unintended effects of trial interventions or trial conduct	Page 15/Line 9-Page 16/Line 6	Safety assessment
Auditing	23	Frequency and procedures for auditing trial conduct, if any, and whether the process will be independent from investigators and the sponsor	N/A	N/A

Ethics and dissemination				
Research ethics approval	24	Plans for seeking research ethics committee/institutional review board (REC/IRB) approval	Page 22/ Line 15-18	Ethical Statement Section
Protocol amendments	25	Plans for communicating important protocol modifications (eg, changes to eligibility criteria, outcomes, analyses) to relevant parties (eg, investigators, REC/IRBs, trial participants, trial registries, journals, regulators)	Page 16/Line 8- Page 17/Line 7	Study quality control and data management
Consent or assent	26a	Who will obtain informed consent or assent from potential trial participants or authorised surrogates, and how (see Item 32)	Page 8/Line 16 - Page 9/Line 7	Randomization and informed consent
	26b	Additional consent provisions for collection and use of participant data and biological specimens in ancillary studies, if applicable	N/ A	N/ A
Confidentiality	27	How personal information about potential and enrolled participants will be collected, shared, and maintained in order to protect confidentiality before, during, and after the trial	Page 16/Line 8- Page 17/Line 7	Study quality control and data management
Declaration of interests	28	Financial and other competing interests for principal investigators for the overall trial and each study site	Page 23/Line 9-11	Funding
Access to data	29	Statement of who will have access to the final trial dataset, and disclosure of contractual agreements that limit such access for investigators	Page 16/Line 8- Page 17/Line 7	Study quality control and data management
Ancillary and post-trial care	30	Provisions, if any, for ancillary and post-trial care, and for compensation to those who suffer harm from trial participation	Page 15/Line 9-Page 16/Line 6	Safety assessment
Dissemination policy	31a	Plans for investigators and sponsor to communicate trial results to participants, healthcare professionals, the public, and other relevant groups (eg, via publication, reporting in results databases, or other data sharing arrangements), including any publication restrictions	N/A	N/A
	31b	Authorship eligibility guidelines and any intended use of professional writers	N/A	N/A
	31c	Plans, if any, for granting public access to the full protocol, participant-level dataset, and statistical code	N/A	N/A
**Appendices**				
Informed consent materials	32	Model consent form and other related documentation given to participants and authorised surrogates	See additional files	See additional files
Biological specimens	33	Plans for collection, laboratory evaluation, and storage of biological specimens for genetic or molecular analysis in the current trial and for future use in ancillary studies, if applicable	N/A	N/A

*It is strongly recommended that this checklist be read in conjunction with the SPIRIT 2013 Explanation & Elaboration for important clarification on the items. Amendments to the protocol should be tracked and dated. The SPIRIT checklist is copyrighted by the SPIRIT Group under the Creative Commons "Attribution-NonCommercial-NoDerivs 3.0 Unported" license.

Article information: http://dx.doi.org/10.21037/apm-20-1418
*As the checklist was provided upon initial submission, the page number/line number reported may be changed due to copyediting and may not be referable in the published version. In this case, the section/paragraph may be used as an alternative reference.

CONSORT 2010 checklist of information to include when reporting a randomised trial*

Section/Topic	Item No	Checklist item	Reported on page No
**Title and abstract**			
	1a	Identification as a randomised trial in the title	Page 1
	1b	Structured summary of trial design, methods, results, and conclusions (for specific guidance see CONSORT for abstracts)	Page 2-3
**Introduction**			
Background and objectives	2a	Scientific background and explanation of rationale	Page 3-5
	2b	Specific objectives or hypotheses	Page 5
**Methods**			
Trial design	3a	Description of trial design (such as parallel, factorial) including allocation ratio	Page 5,6,8
	3b	Important changes to methods after trial commencement (such as eligibility criteria), with reasons	N/A
Participants	4a	Eligibility criteria for participants	Page 7-8
	4b	Settings and locations where the data were collected	Page 5
Interventions	5	The interventions for each group with sufficient details to allow replication, including how and when they were actually administered	Page 9-13
Outcomes	6a	Completely defined pre-specified primary and secondary outcome measures, including how and when they were assessed	Page 13-15
	6b	Any changes to trial outcomes after the trial commenced, with reasons	N/A
Sample size	7a	How sample size was determined	Page 17
	7b	When applicable, explanation of any interim analyses and stopping guidelines	N/A
Randomisation:			
Sequence generation	8a	Method used to generate the random allocation sequence	Page 8,9
	8b	Type of randomisation; details of any restriction (such as blocking and block size)	Page 8,9
Allocation concealment mechanism	9	Mechanism used to implement the random allocation sequence (such as sequentially numbered containers), describing any steps taken to conceal the sequence until interventions were assigned	Page 8,9
Implementation	10	Who generated the random allocation sequence, who enrolled participants, and who assigned participants to interventions	Page 8,9
Blinding	11a	If done, who was blinded after assignment to interventions (for example, participants, care providers, those	Page 9

		assessing outcomes) and how	
	11b	If relevant, description of the similarity of interventions	N/A
Statistical methods	12a	Statistical methods used to compare groups for primary and secondary outcomes	Page 17-18
	12b	Methods for additional analyses, such as subgroup analyses and adjusted analyses	Page 17-18
**Results**			
Participant flow (a diagram is strongly recommended)	13a	For each group, the numbers of participants who were randomly assigned, received intended treatment, and were analysed for the primary outcome	N/A
	13b	For each group, losses and exclusions after randomisation, together with reasons	N/A
Recruitment	14a	Dates defining the periods of recruitment and follow-up	Table 1
	14b	Why the trial ended or was stopped	N/A
Baseline data	15	A table showing baseline demographic and clinical characteristics for each group	N/A
Numbers analysed	16	For each group, number of participants (denominator) included in each analysis and whether the analysis was by original assigned groups	N/A
Outcomes and estimation	17a	For each primary and secondary outcome, results for each group, and the estimated effect size and its precision (such as 95% confidence interval)	N/A
	17b	For binary outcomes, presentation of both absolute and relative effect sizes is recommended	N/A
Ancillary analyses	18	Results of any other analyses performed, including subgroup analyses and adjusted analyses, distinguishing pre-specified from exploratory	N/A
Harms	19	All important harms or unintended effects in each group (for specific guidance see CONSORT for harms)	N/A
**Discussion**			
Limitations	20	Trial limitations, addressing sources of potential bias, imprecision, and, if relevant, multiplicity of analyses	Page 21
Generalisability	21	Generalisability (external validity, applicability) of the trial findings	N/A
Interpretation	22	Interpretation consistent with results, balancing benefits and harms, and considering other relevant evidence	N/A
**Other information**			
Registration	23	Registration number and name of trial registry	Page 3
Protocol	24	Where the full trial protocol can be accessed, if available	N/A
Funding	25	Sources of funding and other support (such as supply of drugs), role of funders	Page 23

*We strongly recommend reading this statement in conjunction with the CONSORT 2010 Explanation and Elaboration for important clarifications on all the items. If relevant, we also recommend reading CONSORT extensions for cluster randomised trials, non-inferiority and equivalence trials, non-pharmacological treatments, herbal interventions, and pragmatic trials. Additional extensions are forthcoming: for those and for up to date references relevant to this checklist, see www.consort-statement.org.
