# Supplementary figures and images for: Efficacy and safety comparison of infrared laser moxibustion and traditional moxibustion in knee osteoarthritis: study protocol for a Zelen-design randomized controlled non-inferiority clinical trial
Source: J Orthop Surg Res. 2023 Dec 2;18:922. doi: 10.1186/s13018-023-04408-x (PMC10693696; doi:10.1186/s13018-023-04408-x)

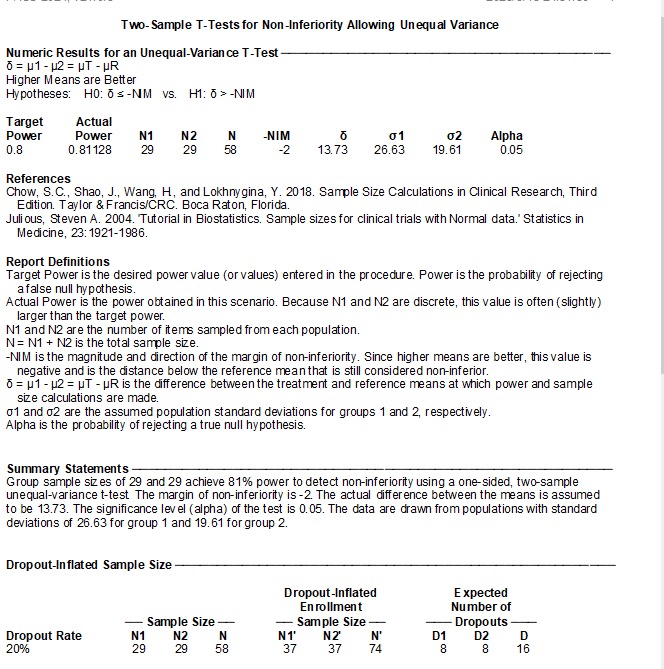

Supplement: Supplementary file 2 — Additional file 2. Sample size calculation. [file 13018_2023_4408_MOESM2_ESM.jpg]
